# Supplementary material for: Cross-cultural validation of two scales to assess mental wellbeing in persons affected by leprosy in Province 1 and 7, Nepal
Source: PLOS Glob Public Health. 2024 Jan 25;4(1):e0002654. doi: 10.1371/journal.pgph.0002654 (PMC10810443; doi:10.1371/journal.pgph.0002654)
Supplement: S1 Checklist — (DOCX) [file pgph.0002654.s001.docx]

STROBE Statement—checklist of items that should be included in reports of observational studies

|  | Item No. | Recommendation | Page  No. | Relevant text from manuscript |
| --- | --- | --- | --- | --- |
| **Title and abstract** | 1 | (*a*) Indicate the study’s design with a commonly used term in the title or the abstract | 1 | Cross-cultural validation study design |
|  |  | (*b*) Provide in the abstract an informative and balanced summary of what was done and what was found | 3 | See Author summary |
| Introduction | | | |  |
| Background/rationale | 2 | Explain the scientific background and rationale for the investigation being reported | 4,5 | See Introduction: “Leprosy is a global health problem […]. In fact, the mental impact is often more severe than the physical effects. […] In Nepal, as “mental illnesses are on the rise” (30), the Nepal Health Research Council acknowledged “the need for a mental health survey” (31). This study discusses two instruments to contribute to meeting this need, suitable for use with vulnerable groups like leprosy-affected persons.” |
| Objectives | 3 | State specific objectives, including any prespecified hypotheses | 5 | “The objective of this study was to test the cultural validity of the Warwick-Edinburgh Mental Wellbeing Scale (WEMWBS) and the Patient Health Questionnaire (PHQ-9, depression tool) for use with persons affected by leprosy in Province 1 and 7, Nepal.” |
| Methods | | | |  |
| Study design | 4 | Present key elements of study design early in the paper | 6 | See Study design |
| Setting | 5 | Describe the setting, locations, and relevant dates, including periods of recruitment, exposure, follow-up, and data collection | 6-8, 9-10 | See Study design, Study population, Study sampling, Administration of the scales, and Data collection |
| Participants | 6 | (*a*) *Cohort study*—Give the eligibility criteria, and the sources and methods of selection of participants. Describe methods of follow-up  *Case-control study*—Give the eligibility criteria, and the sources and methods of case ascertainment and control selection. Give the rationale for the choice of cases and controls  *Cross-sectional study*—Give the eligibility criteria, and the sources and methods of selection of participants | 7-8 | See Study sampling |
|  |  | (*b*) *Cohort study*—For matched studies, give matching criteria and number of exposed and unexposed  *Case-control study*—For matched studies, give matching criteria and the number of controls per case | NA |  |
| Variables | 7 | Clearly define all outcomes, exposures, predictors, potential confounders, and effect modifiers. Give diagnostic criteria, if applicable | 8, 10 | See Cultural equivalence testing, and Data analysis |
| Data sources/ measurement | 8* | For each variable of interest, give sources of data and details of methods of assessment (measurement). Describe comparability of assessment methods if there is more than one group | 7, 9, 10 | See Study design, Administration of the scales, and Data collection |
| Bias | 9 | Describe any efforts to address potential sources of bias | 7, 10 | To avoid sampling bias: “Leprosy-affected persons with a range of impairment severity according to the Eye Hand Foot (EHF) score were included (62), since impairment severity was expected to correlate with mental health status.”  Important to mention: validation design, thus “Selection techniques involved purposive and convenience sampling. This is acceptable for a validation study, since the most important characteristic of the sample is known and an adequate diversity in the trait that is being assessed is aimed for.”  To avoid (researcher) confirmation bias: “the final versions of the tools were discussed with an expert panel.” |
| Study size | 10 | Explain how the study size was arrived at | 6 | “This study aimed to include 20 cases for the qualitative part, or until data saturation was reached, […]. The quantitative sample is based on the minimum number of 100 recommended by Terwee et al. (2007).” |

Continued on next page

| Quantitative variables | 11 | Explain how quantitative variables were handled in the analyses. If applicable, describe which groupings were chosen and why | 8 | Cultural equivalence testing: “*Internal consistency* measured how well all the items in a tool were correlated. It explored the correlation of a given item with the sum score, indicating whether they assessed the same construct. The indicator was Cronbach’s α which would be optimal between 0.70 and 0.90 (61). *Construct validity* was assessed by formulating several hypotheses per scale (see Table 1). The instruments were compared with each other and also with the EHF and EMIC-AP score. If 75% or more of hypotheses were confirmed per instrument, the construct validity was supported (61). *Floor and ceiling effects* were considered to be present if 15% or more of the subjects either had the lowest or the highest possible score on the WEMWBS or PHQ-9, indicating low sensitivity at the low or high end of the score (61).” |
| --- | --- | --- | --- | --- |
| Statistical methods | 12 | (*a*) Describe all statistical methods, including those used to control for confounding | 10 | Data analysis: “The mean sum scores were compared between groups by means of a Student’s T-test, and data of the control group were used as a reference group for “normal” mental wellbeing and depression.” |
|  |  | (*b*) Describe any methods used to examine subgroups and interactions | 9 | Cultural equivalence testing: “For *interpretability*, to help readers interpret the scores, the mean and confidence intervals (CI) of the WEMWBS and PHQ-9 were calculated in three subgroups based on age, gender, and EHF score. The control group was used as a reference.” |
|  |  | (*c*) Explain how missing data were addressed |  | Not applicable. |
|  |  | (*d*) *Cohort study*—If applicable, explain how loss to follow-up was addressed  *Case-control study*—If applicable, explain how matching of cases and controls was addressed  *Cross-sectional study*—If applicable, describe analytical methods taking account of sampling strategy | 7 | Male:female ratio roughly 1. |
|  |  | (*e*) Describe any sensitivity analyses |  | Not applicable. |
| Results | | | | |
| Participants | 13* | (a) Report numbers of individuals at each stage of study—eg numbers potentially eligible, examined for eligibility, confirmed eligible, included in the study, completing follow-up, and analysed | 11 | See Participant characteristics. |
|  |  | (b) Give reasons for non-participation at each stage |  | Not applicable. |
|  |  | (c) Consider use of a flow diagram |  | Not applicable. |
| Descriptive data | 14* | (a) Give characteristics of study participants (eg demographic, clinical, social) and information on exposures and potential confounders | 11, 12 | See Participant characteristics, Table 2 and Table 3 |
|  |  | (b) Indicate number of participants with missing data for each variable of interest |  | Not applicable. |
|  |  | (c) *Cohort study*—Summarise follow-up time (eg, average and total amount) |  |  |
| Outcome data | 15* | *Cohort study*—Report numbers of outcome events or summary measures over time |  |  |
|  |  | *Case-control study—*Report numbers in each exposure category, or summary measures of exposure | 18 | See Interpretability and Table 6 |
|  |  | *Cross-sectional study—*Report numbers of outcome events or summary measures |  |  |
| Main results | 16 | (*a*) Give unadjusted estimates and, if applicable, confounder-adjusted estimates and their precision (eg, 95% confidence interval). Make clear which confounders were adjusted for and why they were included | 18, 19 | 95% CI mentioned. No use of confounders. |
|  |  | (*b*) Report category boundaries when continuous variables were categorized | 18, 19 | Category boundaries mentioned. |
|  |  | (*c*) If relevant, consider translating estimates of relative risk into absolute risk for a meaningful time period |  | Not applicable. |

Continued on next page

| Other analyses | 17 | Report other analyses done—eg analyses of subgroups and interactions, and sensitivity analyses | 16 | See Table 4. Item-Total Statistics (Correlation and Cronbach’s alpha if item was deleted). |
| --- | --- | --- | --- | --- |
| Discussion | | | | |
| Key results | 18 | Summarise key results with reference to study objectives | 23 | See Cultural equivalence and validity: “Overall, looking at the overall results of the five equivalences, good cultural equivalence was achieved for both the WEMWBS and PHQ-9 after relatively small modifications. They can therefore be considered culturally valid in the Nepalese context to measure mental wellbeing and depression among persons affected by leprosy. The validated tools may be used to collect data about mental wellbeing of persons affected by leprosy and their family members, based on which interventions can be developed to improve their mental wellbeing.” |
| Limitations | 19 | Discuss limitations of the study, taking into account sources of potential bias or imprecision. Discuss both direction and magnitude of any potential bias | 23-24 | See Limitations |
| Interpretation | 20 | Give a cautious overall interpretation of results considering objectives, limitations, multiplicity of analyses, results from similar studies, and other relevant evidence | 20-22 | See WEMWBS, and PHQ-9 |
| Generalisability | 21 | Discuss the generalisability (external validity) of the study results | 23 | See Cultural equivalence and validity |
| Other information | |  | | |
| Funding | 22 | Give the source of funding and the role of the funders for the present study and, if applicable, for the original study on which the present article is based | 35 | “No funding was available.” |

*Give information separately for cases and controls in case-control studies and, if applicable, for exposed and unexposed groups in cohort and cross-sectional studies.

**Note:** An Explanation and Elaboration article discusses each checklist item and gives methodological background and published examples of transparent reporting. The STROBE checklist is best used in conjunction with this article (freely available on the Web sites of PLoS Medicine at http://www.plosmedicine.org/, Annals of Internal Medicine at http://www.annals.org/, and Epidemiology at http://www.epidem.com/). Information on the STROBE Initiative is available at www.strobe-statement.org.
