# Supplementary material for: Cross-cultural validation of two scales to assess mental wellbeing in persons affected by leprosy in Province 1 and 7, Nepal
Source: PLOS Glob Public Health. 2024 Jan 25;4(1):e0002654. doi: 10.1371/journal.pgph.0002654 (PMC10810443; doi:10.1371/journal.pgph.0002654)
Supplement: S1 Table — (DOCX) [file pgph.0002654.s002.docx]

**S1 Table.** Definitions of categories to assess cultural equivalence.

| Equivalence | Definition |
| --- | --- |
| Conceptual | “When the questionnaire has the same relationship to the underlying concept in both cultures” |
| Semantic | “Concerned with the transfer of meaning across languages, and with achieving a similar effect on respondents in different languages, […] taking into account a number of different types of meaning.” |
| Item | “When items estimate the same parameters on the latent trait being measured and when they are equally relevant and acceptable in both cultures” |
| Operational | “When the elements [a similar questionnaire format, instructions, mode of administration and measurement methods] do not affect the results.” |
| Measurement | “The extent to which the psychometric properties of different language versions of the same instrument are similar, […] primarily in terms of their reliability, responsiveness and construct validity.” |
