# Supplementary material for: Cross-cultural validation of two scales to assess mental wellbeing in persons affected by leprosy in Province 1 and 7, Nepal
Source: PLOS Glob Public Health. 2024 Jan 25;4(1):e0002654. doi: 10.1371/journal.pgph.0002654 (PMC10810443; doi:10.1371/journal.pgph.0002654)
Supplement: S2 Table — (DOCX) [file pgph.0002654.s003.docx]

**S2 Table.** Adaptation of item statements of WEMWBS and PHQ-9 in Nepalese culture.

|  | Original statements | Statement items revised | Items revised to questions |
| --- | --- | --- | --- |
| WEMWBS | | | |
| 1 | I’ve been feeling optimistic about the future | x | Have you been feeling optimistic about the future? |
| 2 | I’ve been feeling useful | x | Have you been feeling useful? |
| 3 | I’ve been feeling relaxed | x | Have you been feeling relaxed? |
| 4 | I’ve been feeling interested in other people | I’ve been feeling interested in other people (such as family, relatives, neighbours and friends) | Have you been feeling interested in other people (such as family, relatives, neighbours and friends)? |
| 5 | I’ve had energy to spare | x | Have you had energy to spare? |
| 6 | I’ve been dealing with problems well | x | Have you been dealing with problems well? |
| 7 | I’ve been thinking clearly | x | Have you been thinking clearly? |
| 8 | I’ve been feeling good about myself | x | Have you been feeling good about yourself? |
| 9 | I’ve been feeling close to other people | x | Have you been feeling close to other people? |
| 10 | I’ve been feeling confident | I’ve been feeling sure of myself | Have you been feeling sure of yourself? |
| 11 | I’ve been able to make up my own mind about things | x | Have you been able to make up your own mind about things? |
| 12 | I’ve been feeling loved | x | Have you been feeling loved? |
| 13 | I’ve been interested in new things | x | Have you been interested in new things? |
| 14 | I’ve been feeling cheerful | x | Have you been feeling cheerful? |
|  | | | |
| PHQ-9 | | | |
| 1 | Little interest or pleasure in doing things | Little interest or pleasure in doing things/”any work” | Have you been having little interest or pleasure in doing things/”any work”? |
| 2 | Feeling down, depressed or hopeless | x | Have you been feeling down, depressed or hopeless? |
| 3 | Trouble falling asleep, staying asleep, or sleeping too much | x | Have you been having troubles falling asleep, staying asleep, or sleeping too much? |
| 4 | Feeling tired or having little energy | x | Have you been feeling tired or have you been having little energy? |
| 5 | Poor appetite or overeating | x | Have you been having a poor appetite or have you been overeating? |
| 6 | Feeling bad about yourself – or that you’re a failure or have let yourself or your family down | x | Have you been feeling bad about yourself - or that you’re a failure or have let yourself or your family down? |
| 7 | Trouble concentrating on things, such as reading the newspaper or watching television | Trouble concentrating on things, such as reading the newspaper, watching television or listening to music | Have you been having troubles in concentrating on things, such as reading the newspaper, watching television or listening to music? |
| 8 | Moving or speaking so slowly that other people could have noticed. Or, the opposite, being so fidgety or restless that you have been moving around a lot more than usual | x | Have you been moving or speaking so slowly that other people could have noticed. Or, the opposite, have you been so fidgety or restless that you have been moving around a lot more than usual? |
| 9 | Thoughts that you would be better off dead or of hurting yourself in some way | Thoughts of hurting yourself in some way or that you would be better off dead | Have you been having thoughts of hurting yourself in some way or that you would be better off dead? |
| 10 | If you checked of any problems, how difficult have these problems made it for you to: Do your work, take care of things at home, or get along with other people? | x | x |
