# Supplementary material for: Cross-cultural validation of two scales to assess mental wellbeing in persons affected by leprosy in Province 1 and 7, Nepal
Source: PLOS Glob Public Health. 2024 Jan 25;4(1):e0002654. doi: 10.1371/journal.pgph.0002654 (PMC10810443; doi:10.1371/journal.pgph.0002654)
Supplement: S1 Fig — (PDF) [file pgph.0002654.s004.pdf]

S3 Figure. Final WEMWBS and PHQ-9 questionnaires in Nepali language.

भावना र विचारहरुको बारेमा तल केही कथनहरु छन । (WEMWBS)

कृपया बाक्सामा (√) टीक लगाउनुस जस्ले तपाईको **विगत २ हप्ताको** अनुभवलाई बयान गर्दछ ।

कथनहरु	कहिले पनि होईना	शायद नै (१-२ पटक)	बेला बेलामा	प्राय	सधै
१. म मेरो भविष्यको बारेमा आशावादी महसूस गरिरहेको छु	१	२	३	४	५
२. म आफुलाई उपयोगी महसूस गरिरहेको छु	१	२	३	४	५
३. म तनावमुक्त महसूस गरिरहेको छु	१	२	३	४	५
४. म अन्य व्यक्तिहरुमा पनि चासो राखिरहेको छु (जस्तै परिवार, आफन्तहरू, छिमेकीहरू र साथीहरू)	१	२	३	४	५
५. म उर्जाशिल छु	१	२	३	४	५
६. म समस्याहरुको राम्ररी सामना गरिरहेको छु	१	२	३	४	५
७. म स्पष्ट रुपमा सोचिरहेको छु	१	२	३	४	५
८. म आफ्नो बारेमा राम्रै महसूस गरिरहेको छु	१	२	३	४	५
९. म अरु मानिसहरुसित नजिकै भएको महसूस गरिरहेको छु	१	२	३	४	५
१०. म आफैमा बिश्वास भएको महसूस गरिरहेको छु	१	२	३	४	५
११. म आफ्नो विचार बनाउनको लागि सक्षम छु	१	२	३	४	५
१२. मलाई अरुले माया गरेको महसूस गरिरहेको छु	१	२	३	४	५
१३. म नयाँ कुरामा अभिरुचि राख्छु	१	२	३	४	५
१४. म हर्षित महसूस गरिरहेको छु	१	२	३	४	५

बिरामी स्वास्थ्य प्रश्नावली (PHQ-9)

**बिगतदुई हप्ताभित्रमा, कति पटक निम्नलिखितसमस्याहरु मध्ये कुनै पनि समस्याहरुको सामनागर्नु पऱ्यो ?**	**केहि छैन**	**कहिले काही**	**आधादिनभन्दा बढी**	**दिनदिननै**
१. कुनै पनि काम गर्नमा रुची वा खुशीमा कमी	०	१	२	३
२. उदासी, निरासा वा हिनबोध	०	१	२	३
३. निदाउन कठिन, अनिदो, वा धैरे निदाउने	०	१	२	३
४. थकित महशुस वा जाँगरमा कमी	०	१	२	३
५. भोकमा कमी वा धैरे खाने	०	१	२	३
६. आफैलाई खराब महसूस गर्न – वा असफल वा आफैले हिनबोध वा परिवारलाई हिनबोध गराएको ठान्न	०	१	२	३
७. एकाग्रतामा गाह्रो, जस्तै पत्रिका पढन, टिभी हेर्न वा संगीत सुन्न	०	१	२	३
८. विस्तारै हिडेको र बोलेको कुरा अरु मानिसहरुलाई मस्किलले थाहाहुनसक्थ्यो । वा यस्को बिपरीत-अस्वभाविक रुपमा बेचैन वा छटपटिका साथयत्रतत्र हिडन	०	१	२	३
९. कुनै तरीका बाट चोट पुऱ्याउने बिचार आउनु वा यो भन्दा राम्रो मर्नु विचार आउनु	०	१	२	३

जम्मा ....+...........+...........

जम्माजोड ....

१०. यदि तपाईमा उपरोक्त मध्ये कुनै समस्या देखा परयो भने, त्यसले तपाईको काममा, घरमा सरसमानको रेखदेख, वा अरु मानिससँग ब्यवहार गर्नमा समस्या पर्‍यायो ।

☐ कुनै समयस्या छैन ☐ केहि हदसम्म समयस्या छ ☐ धेरै समयस्या छ ☐ अत्यन्तै समयस्या छ
