# Supplementary material for: Cross-cultural validation of two scales to assess mental wellbeing in persons affected by leprosy in Province 1 and 7, Nepal
Source: PLOS Glob Public Health. 2024 Jan 25;4(1):e0002654. doi: 10.1371/journal.pgph.0002654 (PMC10810443; doi:10.1371/journal.pgph.0002654)
Supplement: S1 Text — (DOCX) [file pgph.0002654.s006.docx]

Supplementary material – Semi-structured interviews

Participant quotes of qualitative study

Introductory questions about leprosy:

- No. 2 (F, 25) *“My husband works, he builds homes. He comes home once a month. I said I have leprosy now. I am afraid that he will leave me and marry to another due to leprosy. I haven’t told my parents, daughter or mother-in-law.”*
- No. 4 (F, 24) *“Sometimes I feel different. If other people know about my condition, they may insult me. I belief so.”*
- No. 12 (M, 34) *“[Having been affected by leprosy] It has affected my life. I cannot do my work as I did before. It affects me and my family. I am the main guardian of the family. I am suffering from leprosy so my family members are facing problems such as economic problems.”*

WEMWBS

1. **I’ve been feeling optimistic about the future**

Related to optimism (WEMWBS: Statement 1 and 14)

1. **I’ve been feeling useful**

Related to usefulness (WEMWBS: Statement 2; PHQ-9: Statement 10)

1. **I’ve been feeling relaxed**

Related to feeling relaxed/tense (WEMWBS: Statement 3; PHQ-9: Statement 8)

- No. 2 (F, 25) *“Yes, some of the time I feel relaxed. But sometimes I think; how did leprosy start with me? I think because I used the powder, or I did the facial. Don’t do a facial or use the powder at night.”* [health/disease, fear]
- No. 12 (M, 34) *“Often I am feeling relaxed. […] But sometimes I think of the fact that I am affected by leprosy now, and I cannot do anything about it. But then after a few minutes I will be ok and say that it’s nothing and I will be fine.”* [health/disease]

1. **I’ve been feeling interested in other people, such as family, relatives, neighbours and friends**

Related to interest (WEMWBS: Statement 4 and 13; PHQ-9: Statement 1)

1. **I’ve had energy to spare**

Related to energy (WEMWBS: Statement; PHQ-9: Statement 3 and 4)

- No. 14 (M, 28) *laughs* *“I don’t do a lot of work in my home. I only clean my room, that is my daily work. I don’t do anything else, because my mother and sister in law are there in home, so most of the time I will be busy with studying.”*

1. **I’ve been dealing with problems well**

Related to decision-making (WEMWBS: Statement 6, 7 and 11)

- No. 5 (F, 15) *“Rarely I will deal with the problems. Most of the time I take help from sister and my family.”* [gender, dependence, position in family]
- No. 1 (F, 32) *“I alone cannot deal with the problem. Sometimes I take help with him.”* [gender, dependence, position in family]
- No. 3 (F, 50) *“If I have a small problem, rarely I deal with them. But all of the time I will talk with my sons and husband for dealing with problems.”*
- No. 4 (F, 24) *“Some of the time I can deal with problems, and sometimes I take help from friends and family members […].”*
- No. 13 (M, 38) “*My mother is old, so now I am the head of my family. I must have to deal with all the problems. Often I am dealing with the problems and sometimes I take help from my wife and my parents too."* [position in family]

1. **I’ve been thinking clearly**

Related to decision-making (WEMWBS: Statement 6, 7 and 11)

- No. 1 (F, 32) *“I have disease and a problem. I don’t have a child so also due to this I rarely think clearly. Rest of the time I don’t think clearly.”* [health/disease, family]
- No. 3 (F, 50) *“I don’t think clearly because I am uneducated and physically not well. My family members do all the things and what I need they bring for me, so I don’t have to think about anything anymore.”* [health/disease, education, gender, position in family]
- No. 6 (F, 41) *“Rarely, all of the people help me so I don’t have to think anything.”* [dependence, respect]
- No. 15 (M, 45) *“Up to today I have always been thinking clearly. If I don’t feel good, then I don’t think clearly.”* [health/disease]

1. **I’ve been feeling good about myself**

Related to confidence (WEMWBS: Statement 8, 10 and 11; PHQ-9: Statement 6)

- No. 13 (M, 38) *“I have been feeling good all the time. I don’t feel bad about my leprosy, because it will be cured after the treatment.”* [health]
- No. 15 (M, 45) “*Often I feel good about myself. If the work I did is going in the right direction, then I feel good.”* [work]

1. **I’ve been feeling close to other people**

Related to respect (WEMWBS: Statement 9, 12 and 14; PHQ-9: Statement 10)

- No. 3 (F, 50) *“I am often close to everyone but they don’t know about my condition. So they are close but if I tell them about the disease, they may be far away from me. They may treat me in bad way.”* [health/disease]

1. **I’ve been feeling sure of myself**

Related to confidence (WEMWBS: Statement 8, 10 and 11; PHQ-9: Statement 6)

- No. 3 (F, 50) *“I am confident all the time because I am taking the medicine. I trust the doctor and he told me that if there is any problem, I must come here. So if I have a problem, I will come back here again.”* [health/disease]
- No. 5 (F, 18) *“I am confident. Often when I felt sick and I was not with my family; at that time I don’t feel confident.”* [health/disease]
- No. 6 (F, 41) *“All of the time I am feeling confident, but I am not confident about my daughter what she will do in the future. […]”* [family]
- No. 12 (M, 34) *“I have been feeling confident all of the time. When my family, my relatives and my friends are supportive, then I feel confident.”* [family, respect]
- No. 13 (M, 38) *“All of the time I am confident. [I feel confident] When all of the helping hands are with me. [I don’t feel confident] If I would get sick or if I would get an accident.”* [respect, health/disease]

1. **I’ve been able to make up my own mind about things**

Related to decision-making (WEMWBS: Statement 6, 7 and 11), and related to confidence (WEMWBS: Statement 8, 10 and 11; PHQ-9: Statement 6)

- No. 1 (F, 32) *“Sometimes I am able to make up my own mind about things. But most of the time my husband makes decisions and I must have to follow him.”* [gender, position in family]
- No. 4 (F, 24) *“Sometimes I have to take help from someone, and some of the time I am able to make up my mind myself.”*

1. **I’ve been feeling loved**

Related to respect (WEMWBS: Statement 9, 12 and 14; PHQ-9: Statement 10)

1. **I’ve been interested in new things**

Related to interest (WEMWBS: Statement 4 and 13; PHQ-9: Statement 1)

- No. 2 (F, 25) *“I am not interested in new things. I will do my own work. I will grow vegetables and see it to the market.”* [work]

1. **I’ve been feeling cheerful**

Related to optimism (WEMWBS: Statement 1 and 14), and related to respect (WEMWBS: Statement 9, 12 and 14; PHQ-9: Statement 10)

PHQ-9

1. **Little interest or pleasure in doing things**

Related to interest (WEMWBS: Statement 4 and 13; PHQ-9: Statement 1)

- No. 13 (M, 38) *“Not at all, I will be in my shop every day for the whole day.”* [work]
- No. 14 (M, 28) *“Not at all, there is no more work in my home. I must have to clean my room and wash my clothes, but sometimes when I am alone I don’t like to work.”*

1. **Feeling down, depressed or hopeless**

Related to feeling down, sad and/or hopeless (PHQ-9: Statement 2 and 9)

- No. 1 (F, 32) *“I don´t feel down or depressed, because I am busy in my work.”*
- No. 4 (F, 24) *“Several days I feel down and hopeless if I cannot complete my work in time […].”* [work load]
- No. 12 (M, 34) *“Several days I feel down. If I am alone, I think about how it [having leprosy] happened to me. I used to be hopeless, that I could not do good, so I always want to be busy in doing things.”* [health/disease, fear]

1. **Trouble falling asleep, staying asleep, or sleeping too much**

Related to energy (WEMWBS: Statement; PHQ-9: Statement 3 and 4)

- No. 12 *“I always sleep well. Nowadays the nights are short, so I sleep 1 hour during the day.”* [sleep]

1. **Feeling tired or having little energy**

Related to energy (WEMWBS: Statement; PHQ-9: Statement 3 and 4)

- No. 1 (F, 32) *“Several days I feel tired by doing my work. There is no one to help me. I have a husband but he goes to work.”* [work load]

1. **Poor appetite or overeating**

Related to appetite (PHQ-9: Statement 5)

- No. 4 (F, 24) *“I don’t eat less but when I sit in my shop then I eat more fast food. But I eat rice in morning and at night.”*
- No. 5 (F, 18) *“Several days I had poor appetite due to climatic condition or due to warmer day.”* [climate]
- No. 12 (M, 34) *“In the last two months I am eating more than before. I think this may be due to medicine and my working load.”* [medicine, work load]

1. **Feeling bad about yourself – or that you’re a failure or have let yourself or your family down**

Related to confidence (WEMWBS: Statement 8, 10 and 11; PHQ-9: Statement 6)

- No. 12 (M, 34) *“Sometimes I feel bad about myself because if I was not suffering from leprosy, I would be working abroad to earn money. I can earn more abroad than here.”* [health/disease, work, responsibility]

1. **Outside your routine work: Trouble concentrating on things, such as reading the newspaper, watching television or listening to music**

Related to concentration (PHQ-9: Statement 7)

1. **Moving or speaking so slowly that other people could have noticed. Or, the opposite – being so fidgety or restless that you have been moving around a lot more than usual**

Related to feeling relaxed/tense (WEMWBS: Statement 3; PHQ-9: Statement 8)

- No. 1 *“Several days people notice me.” “Why?” “I don’t know, but they look at me.” “Did they know about your leprosy?” “I didn’t tell to them. Only my parents, and my husband parents know about that.”* [feeling tension]

1. **Thoughts of hurting yourself in some way or that you would be better off dead**

Related to feeling down, sad and/or hopeless (PHQ-9: Statement 2 and 9)

- No. 1 (F, 32) *“Sometimes I like hurting myself.” “Why?” “I don´t have a baby, and I got a disease leprosy too. And I will walk in home and I am free I think about that. I don’t have a child too. My husband goes to work, and my mother in law says I don’t have a baby. She said: I will marry another girl to my son. I am together with my husband for 10 years but we don’t have a child.”* [disease, marriage]
- No. 4 (F, 24) *“Several days I have had thoughts about hurting myself. This is because sometimes my parents scold me that I didn’t do household activities, so I want to hurt myself.”* [family, responsibility]
- No. 6 (F, 41) *“Several days I thoughts of hurting myself. If I take medicine and it won’t be cured? Then I thought about hurting myself.”* [fear]

1. **If you checked off any problems, how difficult have those problems made if for you to do your work, take care of things at home, or get along with other people?**

Related to usefulness (WEMWBS: Statement 2; PHQ-9: Statement 10), and related to respect (WEMWBS: Statement 9, 12 and 14; PHQ-9: Statement 10)
