# Supplementary material for: Cross-cultural validation of two scales to assess mental wellbeing in persons affected by leprosy in Province 1 and 7, Nepal
Source: PLOS Glob Public Health. 2024 Jan 25;4(1):e0002654. doi: 10.1371/journal.pgph.0002654 (PMC10810443; doi:10.1371/journal.pgph.0002654)
Supplement: Attachment — Submitted filename: Reviewer Feedback on the Manuscript CrossCultural Validation of Two Scales to Assess Mental Health in Leprosy.pdf [file pgph.0002654.s008.pdf]

Reviewer's Feedback on the Manuscript: Cross-Cultural Validation of Two Scales to Assess Mental Health in Leprosy-Affected Persons in Province 1 and 7, Nepal

The Editor,

Thank you very much for the opportunity to review this manuscript.

The paper was about assessing the Cross-Cultural validity of the Warwick-Edinburgh Mental Wellbeing Scale (WEMWBS) and the Patient Health Questionnaire (PHQ-9) among persons affected by leprosy in Province 1 and 7 of Nepal. The study tools' validity was ascertained by 5 indices of equivalence: conceptual, semantic, item, operational and measurement equivalence. This is an important work as the validated tools provides a means to accurately conduct culturally appropriate research, fostering a deeper understanding of nuances among persons affected by leprosy in Nepal in this sensitive area of mental health and wellbeing.

The study objectives were clear, methodology was well-articulated with appropriate study design and description of the study population with sufficient sample size. The result section was clearly presented with sufficient tables to answer the study objectives. The data presented in this study supports the conclusion. The authors additionally stated the public health relevance of their findings.

It my opinion that the manuscript should be accepted for publication, after due considerations of the minor issues highlighted for revision as aforementioned. Thank you.

*****

Thanks to the authors for conducting this important research to develop a translated and validated version of the WEMWBS and PHQ-9 tools for use among persons affected by leprosy in the Nepalese population.

The study objectives were clear, methodology was well-articulated with appropriate study design and description of the study population with sufficient sample size. The result section was clearly presented with sufficient tables to answer the study objectives. The data presented in this study supports the conclusion.

However, I have some comments for your consideration.

General comments

Kindly change the phrase "leprosy-affected persons". May be better to use a non-discriminatory terminology like "persons affected by leprosy" which is applicable to any other condition.

Author's summary

"…the validated tools can be used to quantify the double stigma of leprosy and mental wellbeing in Nepal,…". What do you mean by double stigma of mental wellbeing? Kindly rephrase to convey intended meaning or context.

Introduction

1. WEMWBS and PHQ-9: "A score of 40 or below suggests low mental wellbeing (37)."
   Kindly recheck/confirm as quoted figure and interpretation seems at variance with citation. For instance, do you refer to a 'mean score' of 40? When used categorically, is a single absolute value used as a cut-off or as a range of the mean score with the standard deviation?
   Also, may be more appropriate to cite the actual WEMWBS guide recommended for scoring, analysing and interpreting the tool.
2. "The Explanatory Model Interview Catalogue Stigma Scale for Affected Persons (EMIC-AP) is an existing stigma measure that has been validated in…"
   Kindly provide a brief explanation here as to why you are introducing the EMIC-AP tool e.g., for the purpose of assessing the construct validity of the tools, etc.

Methods

1. Study population: "The quantitative sample is based on the minimum number of 100 recommended by Terwee et al. (2007)."
   Please use proper citation style.
2. Correct tenses to read: "*Internal consistency* measured how well all the items in the tool was correlated."
3. Data collection: "The semi-structured interviews were audio recorded. After revision of the tools, structured interviews were used to test the psychometric properties of the scales."
   For clarity, kindly replace "structured interviews". E.g., may be better to clearly state interviewer-administered questionnaire to distinguish from the qualitative component (semi-structured interviews). Make similar correction across entire document.

4. Data analysis: The first 2 sentences of this section differ from the stated method above using the WHO translation guideline. If the 4-step forward translation, pretesting, etc approach was used, summarize accordingly for the qualitative data.
5. Data analysis: Description of quantitative data analysis to assess measurement equivalence is appropriate. However, last sentence on statistical significance may not be relevant.
6. Ethical considerations: On last sentence for compensation for lost wages; Amount given should be stated, if given.

Results

1. Twenty cases: reads better as Twenty respondents. Take note of importance of sensitivity to language.
2. "The study population shows a range of age (18 to 63; median 33)": Would read better as: Median age was 33 years (range 18 to 63 years) and _% attained __ as highest level of education (or rephrase as appropriate).
3. Interpretability: "The cut-off value suggesting "good wellbeing" is above 40." Kindly refer to above comment on cut-off value of WEMWBS interpretation.
4. Interpretability: "These findings suggest a slightly poorer mental wellbeing among cases than controls."
   Within this context, these findings (mean scores + confidence intervals) suffice to ascertain interpretability. I do not agree that it also suggests generalization about the wellbeing within any of the study groups which were purposively and conveniently sampled, not randomized. I suggest this sentence should be removed.
5. Interpretability: "These findings also suggest a slightly poorer mental wellbeing among cases than controls." Please see comment on similar statement above.

Discussions

1. "All in all, the WEMWBS is a good alternative to the quality of life instruments…"
   This is a bit unclear though the citations imply a direct reference/comparison with the WHOQOL-BREF; please clarify. If yes however, this does not speak to the study objective so may not be an appropriate conclusion here, based on this study results. Furthermore, while I agree that both tools share similarities, it is not convincing that they are alternatives, as their use should depend on the intended objective or choice of outcome measure.
2. PHQ-9: "Scores of 10-14 are indicative of moderately severe depression and hence that people need mental health care help that may be given in the community, preferably using psychological treatment methods. People with severe depression should be referred for professional mental health care."
   Not relevant to this study's objective. Please see similar comment above on generalization.

References

Reference #2: Please check and include a valid link.
