# Supplementary material for: Cross-cultural validation of two scales to assess mental wellbeing in persons affected by leprosy in Province 1 and 7, Nepal
Source: PLOS Glob Public Health. 2024 Jan 25;4(1):e0002654. doi: 10.1371/journal.pgph.0002654 (PMC10810443; doi:10.1371/journal.pgph.0002654)
Supplement: Attachment — Submitted filename: 231022_ResponsetoReviewers.docx [file pgph.0002654.s009.docx]

# **Manuscript PGPH-D-23-01434**

# **Response to Reviewers**

Dear Dr. Preeti Mahato,

Academic Editor of PLOS Global Public Health,

Thank you for giving us the opportunity to submit a revised draft of the manuscript “Cross-Cultural Validation of Two Scales to Assess Mental Wellbeing in Persons Affected by Leprosy in Province 1 and 7, Nepal” for publication in the journal PLOS Global Public Health. We appreciate the time and effort that you and the reviewers dedicated to providing feedback on our manuscript and are grateful for the insightful comments on and valuable improvements to our paper. We have incorporated most of the suggestions made by the reviewers. Those changes are highlighted within the manuscript. Please see below, in blue, for a point-by-point response to the reviewers’ comments and concerns. All page numbers refer to the revised manuscript file with tracked changes.

Best regards,

Janna Dijkstra

Nov 2023

**Editor/Reviewer’s Feedback on the Manuscript: Cross-Cultural Validation of Two Scales to Assess Mental** **Health in Leprosy-Affected Persons in Province 1 and 7, Nepal**

**Academic Editor**

Thank you very much for this important study on cross-cultural validation of two scales to assess mental health in leprosy affected people in Nepal. Mental well-being of leprosy affected people is very important due to stigma related to leprosy in countries such as Nepal. Please work on the comments provided by both reviewers. Here are few of my own observations.

**Author response**: Thank you!

I wonder if the title could contain the word mental well-being than mental health as assessing mental well-being of these patients was mentioned in the background and rationale for this study.

**Author response**: Thank you for this suggestion. The editor is correct, and we have changed the title accordingly.

You mentioned in the objective of the study 'to test the cultural validity of the Warwick-Edinburgh Mental Wellbeing Scale (WEMWBS) and the Patient Health Questionnaire (PHQ-9, depression tool) for use with persons affected by leprosy'. These scales can also be used to assess mental well-being among people suffering from other diseases such as HIV which also has an element of stigma. Although in the last concluding sentence on future research using these scales was suggested in lymphatic filariasis and other neglected tropical diseases, I was curious if it would also be applicable to disease such as HIV. This is my personal opinion/recommendation as I have done some research on stigma related to HIV, but not necessarily needs to be addressed in the discussion or conclusion of this study.

**Author response**: Thank you for this suggestion. Although we agree that this is an important consideration, we have not included it in this manuscript mainly because we do not have the knowledge of up-to-date literature nor the experience with HIV-related stigma. However, we will take this into consideration in our following stigma-related studies.

Journal Requirements:

1. Please include a complete copy of PLOS’ questionnaire on inclusivity in global research in your revised manuscript. Our policy for research in this area aims to improve transparency in the reporting of research performed outside of researchers’ own country or community. The policy applies to researchers who have travelled to a different country to conduct research, research with Indigenous populations or their lands, and research on cultural artefacts. The questionnaire can also be requested at the journal’s discretion for any other submissions, even if these conditions are not met. Please find more information on the policy and a link to download a blank copy of the questionnaire here: https://journals.plos.org/globalpublichealth/s/best-practices-in-research-reporting. Please upload a completed version of your questionnaire as Supporting Information when you resubmit your manuscript.

**Author response**: Thank you for pointing this out. Please find attached a completed version of PLOS’ questionnaire on inclusivity in global research.

2. We ask that a manuscript source file is provided at Revision. Please upload your manuscript file as a .doc, .docx, .rtf or .tex.

Please review your reference list to ensure that it is complete and correct. If you have cited papers that have been retracted, please include the rationale for doing so in the manuscript text, or remove these references and replace them with relevant current references. Any changes to the reference list should be mentioned in the rebuttal letter that accompanies your revised manuscript. If you need to cite a retracted article, indicate the article’s retracted status in the References list and also include a citation and full reference for the retraction notice.

**Author response**: Thank you for pointing this out. The following references have been updated:

(2) World Health Organization. Leprosy [Internet]. 2023 [cited 2023 Oct 8]. Available from: <https://www.who.int/news-room/fact-sheets/detail/leprosy>.

(19) Government of Nepal - Ministry of Health & Population. Leprosy Control and Disability Management Section [Internet]. 2022 [cited 2023 Oct 8]. Available from: <http://dohs.gov.np/leprosy-control-and-disability-management-section/>

(31) Dhimal M, Dahal S, Adhikari K, Koirala P, Bista B, Luitel N, et al. A Nationwide Prevalence of Common Mental Disorders and Suicidality in Nepal: Evidence from National Mental Health Survey, 2019-2020. J Nepal Health Res Counc [Internet]. 2022 Mar 13 [cited 2023 Oct 8];19(4):740–7. Available from: <https://pubmed.ncbi.nlm.nih.gov/35615831/>

(35) Warwick Medical School. University of Warwick. 2018 [cited 2023 Oct 8]. WEMWBS in Different Languages. Available from: <https://warwick.ac.uk/fac/sci/med/research/platform/wemwbs/using/translations/>

(37) Warwick WMS. University of Warwick. 2015 [cited 2023 Oct 8]. Warwick-Edinburgh Mental Wellbeing Scale (WEMWBS). Available from: <https://warwick.ac.uk/fac/sci/med/research/platform/wemwbs>

(63) World Health Organization. Process of translation and adaptation of instruments [Internet]. 2018 [cited 2023 Oct 8]. p. 6–7. Available from: <https://terrance.who.int/mediacentre/data/WHODAS/Guidelines/WHODAS%202.0%20Translation%20guidelines.pdf>

Following reference was not available anymore and thus deleted from the reference list:

(80) ChaMPs Public Health Network. Measuring mental wellbeing: Using the Warwick-Edinburgh Mental Wellbeing Scale (WEMWBS) to measure mental wellbeing as an outcome of your intervention [Internet]. [cited 2018 Sep 28]. Available from: [http://www.champspublichealth.com/writedir/2967Measuring mental wellbeing using WEMWBS-FINAL.pdf](http://www.champspublichealth.com/writedir/2967Measuring%20mental%20wellbeing%20using%20WEMWBS-FINAL.pdf)

In addition, eight references were cleaned in the manuscript for publication (i.e., removal of “[Internet]” of “[cited …]”).

**Reviewer 1**

The Editor,

Thank you very much for the opportunity to review this manuscript.

The paper was about assessing the Cross-Cultural validity of the Warwick-Edinburgh Mental Wellbeing

Scale (WEMWBS) and the Patient Health Questionnaire (PHQ-9) among persons affected by leprosy in

Province 1 and 7 of Nepal. The study tools’ validity was ascertained by 5 indices of equivalence: conceptual, semantic, item, operational and measurement equivalence. This is an important work as the validated tools provides a means to accurately conduct culturally appropriate research, fostering a deeper understanding of nuances among persons affected by leprosy in Nepal in this sensitive area of mental health and wellbeing.

The study objectives were clear, methodology was well-articulated with appropriate study design and

description of the study population with sufficient sample size. The result section was clearly presented with sufficient tables to answer the study objectives. The data presented in this study supports the conclusion. The authors additionally stated the public health relevance of their findings.

It my opinion that the manuscript should be accepted for publication, after due considerations of the

minor issues highlighted for revision as aforementioned. Thank you.

*****

Thanks to the authors for conducting this important research to develop a translated and validated version of the WEMWBS and PHQ-9 tools for use among persons affected by leprosy in the Nepalese population. The study objectives were clear, methodology was well-articulated with appropriate study design and description of the study population with sufficient sample size. The result section was clearly presented with sufficient tables to answer the study objectives. The data presented in this study supports the conclusion. However, I have some comments for your consideration.

**Author response**: Thank you!

**General comments**

Kindly change the phrase “leprosy-affected persons”. May be better to use a non-discriminatory terminology like “persons affected by leprosy” which is applicable to any other condition.

**Author response**: Thank you for this suggestion. We agree with the reviewer and have changed “leprosy-affected persons” to “persons affected by leprosy” throughout the manuscript.

**Author’s summary**

“...the validated tools can be used to quantify the double stigma of leprosy and mental wellbeing in Nepal,...”. What do you mean by double stigma of mental wellbeing? Kindly rephrase to convey intended meaning or context.

**Author response**: Thank you for pointing this out. Here we mean the double stigma of both leprosy and poor mental wellbeing: the stigma related to leprosy, and the stigma related to mental health problems. The Author’s Summary has been updated, such that this double stigma is made clearer.

**Introduction**

1. WEMWBS and PHQ-9: “A score of 40 or below suggests low mental wellbeing (37).” Kindly recheck/confirm as quoted figure and interpretation seems at variance with citation. For instance, do you refer to a ‘mean score’ of 40? When used categorically, is a single absolute value used as a cut-off or as a range of the mean score with the standard deviation? Also, may be more appropriate to cite the actual WEMWBS guide recommended for scoring, analysing and interpreting the tool.

**Author response**: Thank you for your comment. We have confirmed the interpretation with two citations. The current phrase in the manuscript is as follows on page 5:

*“A score of 40 or below suggests low mental wellbeing (37,39).”*

Regarding the correct citation, thank you for this suggestion. We chose to link to the main website of WEMWBS, including content on “Collect, score, analyse and interpret WEMWBS”. We did not find a separate scoring guide.

2. “The Explanatory Model Interview Catalogue Stigma Scale for Affected Persons (EMIC-AP) is an existing stigma measure that has been validated in...” Kindly provide a brief explanation here as to why you are introducing the EMIC-AP tool e.g., for the purpose of assessing the construct validity of the tools, etc.

**Author response**: Thank you for this suggestion. To introduce the tool, we have added the following sentence on page 6:

*“To assess the construct validity of the tools, a third tool was used in this study.”*

**Methods**

1. Study population: “The quantitative sample is based on the minimum number of 100 recommended by Terwee et al. (2007).” Please use proper citation style.

**Author response**: Thank you for pointing this out. We have used the proper citation style.

2. Correct tenses to read: “Internal consistency measured how well all the items in the tool was correlated.”

**Author response**: Thank you for your comment. The current phrase in the manuscript is as follows on page 8:

*“Internal consistency measures how well all the items in a tool are correlated.”*

3. Data collection: “The semi-structured interviews were audio recorded. After revision of the tools, structured interviews were used to test the psychometric properties of the scales.” For clarity, kindly replace “structured interviews”. E.g., may be better to clearly state interviewer-administered questionnaire to distinguish from the qualitative component (semi-structured interviews). Make similar correction across entire document.

**Author response**: We agree with the reviewer’s assessment. Accordingly, throughout the manuscript,

we have revised “structured interviews” to “interviewer-administered questionnaires”.

4. Data analysis: The first 2 sentences of this section differ from the stated method above using the WHO translation guideline. If the 4-step forward translation, pretesting, etc approach was used, summarize accordingly for the qualitative data.

**Author response**: Thank you for pointing this out. The steps mentioned in the Methods section are indeed forward translation, pre-testing, an expert panel, pre-testing and back-translation. Please find the revised subsection under “Data analysis” on page 10:

*“The tools were forward translated by the interviewer/translator and pre-tested on participants. The semi-structured interviews were transcribed and translated by the interviewer/translator and analysed by the principal investigator. Using framework analysis and coding, interpretations of the statements were divided into being within or out of the range of what the statement was intended to mean (64). If a majority of participants misinterpreted the question, the items were discussed with an expert panel. Items were revised accordingly and again pre-tested. Before accepting the revised instruments for use in the quantitative study, the final versions of the tools were back translated by an independent translator.”*

5. Data analysis: Description of quantitative data analysis to assess measurement equivalence is appropriate. However, last sentence on statistical significance may not be relevant.

**Author response**: Thank you for pointing this out. Indeed, we do not aim to generalise the findings beyond the samples as the selection of study participants was not random; therefore, the sample was not representative of the whole study population. However, the use of statistical comparison is warranted as part of the hypotheses testing for construct validity.

6. Ethical considerations: On last sentence for compensation for lost wages; Amount given should be stated, if given.

**Author response**: We have added the suggested content to the manuscript on page 11:

*“No incentives were offered other than a bus fare or compensation for lost wages, if appropriate (max. 500 Nepalese rupee).”*

**Results**

1. Twenty cases: reads better as Twenty respondents. Take note of importance of sensitivity to language.

**Author response**: We have added the suggested content to the manuscript on page 11.

2. “The study population shows a range of age (18 to 63; median 33)”: Would read better as: Median age was 33 years (range 18 to 63 years) and _% attained __ as highest level of education (or rephrase as appropriate).

**Author response**: Thank you for pointing this out. The reviewer is correct, and we have changed this sentence. The revised text reads as follows on page 11:

*“Median age was 33 years (range 18-63) and 60% attained secondary or higher education (e.g., university) as highest level of education.”*

3. Interpretability: “The cut-off value suggesting “good wellbeing” is above 40.” Kindly refer to above comment on cut-off value of WEMWBS interpretation.

**Author response**: Thank you for pointing this out. The revised text reads as follows on page 18, including reference:

*“The cut-off value indicative of low mental wellbeing is 40 or below (37).”*

4. Interpretability: “These findings suggest a slightly poorer mental wellbeing among cases than controls.” Within this context, these findings (mean scores + confidence intervals) suffice to ascertain interpretability. I do not agree that it also suggests generalization about the wellbeing within any of the study groups which were purposively and conveniently sampled, not randomized. I suggest this sentence should be removed.

**Author response**: We think this is an excellent suggestion. We have removed the abovementioned sentence on page 18. We have added the following in the Methods section on page 10:

*“The total scores of the WEMWBS and PHQ-9 (and EMIC-AP) for each subject were summed and a mean sum score was used for further analysis.”*

5. Interpretability: “These findings also suggest a slightly poorer mental wellbeing among cases than controls.” Please see comment on similar statement above.

**Author response**: Thank you for pointing this out. We have removed the abovementioned sentence on page 19.

**Discussions**

1. “All in all, the WEMWBS is a good alternative to the quality of life instruments...” This is a bit unclear though the citations imply a direct reference/comparison with the WHOQOL-BREF; please clarify. If yes however, this does not speak to the study objective so may not be an appropriate conclusion here, based on this study results. Furthermore, while I agree that both tools share similarities, it is not convincing that they are alternatives, as their use should depend on the intended objective or choice of outcome measure.

**Author response**: Thank you for your suggestion. We have deleted the reference on WHOQOL-BREF (Skevington, 2004) and revised the sentence on page 21:

*“All in all, the WEMWBS is a good choice for measuring mental wellbeing, especially in a target group with low education (75). In addition, the instrument could be added to quality-of-life assessments, for instance, for persons affected by leprosy (76).”*

2. PHQ-9: “Scores of 10-14 are indicative of moderately severe depression and hence that people need mental health care help that may be given in the community, preferably using psychological treatment methods. People with severe depression should be referred for professional mental health care.” Not relevant to this study’s objective. Please see similar comment above on generalization.

**Author response**: Thank you for pointing this out. Although we agree that this is an important consideration in the Results section, we do believe it is relevant to mention in the Discussion. It refers to a wider applicability of the scale once it has been validated and then used. It puts the scale in further context.

**References**

Reference #2: Please check and include a valid link.

**Author response**: Thank you for pointing this out. The link has been updated and corrected on page 25.

**Reviewer 2**

This study has shown that the researchers already used a strong method, since they used both qualitative and quantitative approach for this cultural adaptation instrument. The number of sample was enough and the statistics method appropriate for to achieve the conclusion.

**Author response**: Thank you!

However, the part of discussion should be revised since most of it only repetition it is only of the result. It is suggested not to mention the same this twice in the manuscript. Discussion should explain more about the culture as local context for the instrument.

**Author response**: Thank you for this suggestion. We agree that this is an important consideration and we have tried to put the scales into the local/cultural perspective. It would have been interesting to explore this aspect more, however due to the already long length of our manuscript, we have decided to not add more content.
